# Cerebral Autoregulation, Cerebral Hemodynamics, and Injury Biomarkers, in Patients with COVID-19 Treated with Veno-Venous Extracorporeal Membrane Oxygenation

**DOI:** 10.1007/s12028-023-01700-w

**Published:** 2023-03-22

**Authors:** Małgorzata Burzyńska, Agnieszka Uryga, Magdalena Kasprowicz, Marek Czosnyka, Waldemar Goździk, Chiara Robba

**Affiliations:** 1 Department of Anaesthesiology and Intensive Care, Wroclaw Medical University, Wroclaw, Poland; 2 Department of Biomedical Engineering, Faculty of Fundamental Problems of Technology, Wroclaw University of Science and Technology, Wybrzeze Wyspianskiego 27, 50-370 Wroclaw, Poland; 3 Brain Physics Laboratory, Division of Neurosurgery, Department of Clinical Neurosciences, Addenbrooke’s Hospital, University of Cambridge, Cambridge, UK; 4 Institute of Electronic Systems, Faculty of Electronics and Information Technology, Warsaw University of Technology, Warsaw, Poland; 5 IRCCS, Ospedale Policlinico San Martino, Genoa, Italy; 6 Department of Surgical Sciences and Integrated Diagnostics, University of Genoa, Viale Benedetto XV 16, Genoa, Italy

**Keywords:** COVID-19, Extracorporeal membrane oxygenation, Phosphopyruvate hydratase, Cerebrovascular circulation

## Abstract

**Background:**

This study aimed to describe the cerebrovascular dynamics, in particular cerebral autoregulation (CA), and cerebral biomarkers as neuron-specific enolase (NSE) in patients with a diagnosis of coronavirus disease 2019 and acute respiratory distress syndrome as well as undergoing veno-venous extracorporeal membrane treatment.

**Methods:**

This was a single center, observational study conducted in the intensive care unit of the University Hospital in Wroclaw from October 2020 to February 2022. Transcranial Doppler recordings of the middle cerebral artery conducted for at least 20 min were performed. Cerebral autoregulation (CA) was estimated by using the mean velocity index (Mxa), calculated as the moving correlation coefficient between slow-wave oscillations in cerebral blood flow velocity and arterial blood pressure. Altered CA was defined as a positive Mxa. Blood samples for the measurement of NSE were obtained at the same time as transcranial Doppler measurements.

**Results:**

A total of 16 patients fulfilled the inclusion criteria and were enrolled in the study. The median age was 39 (34–56) years. Altered CA was found in 12 patients, and six out of seven patients who died had altered CA. A positive Mxa was a significant predictor of mortality, with a sensitivity of 85.7%. We found that three out of five patients with pathological changes in brain computed tomography and six out of ten patients with neurological complications had altered CA. NSE was a significant predictor of mortality (cutoff value: 28.9 µg/L); area under the curve = 0.83, *p* = 0.006), with a strong relationship between increased level of NSE and altered CA, *χ*^2^ = 6.24; *p* = 0.035; φ = 0.69.

**Conclusions:**

Patients with coronavirus disease 2019–related acute respiratory distress syndrome, requiring veno-venous extracorporeal membrane treatment, are likely to have elevated NSE levels and altered CA. The CA was associated with NSE values in this group. This preliminary analysis suggests that advanced neuromonitoring and evaluation of biomarkers should be considered in this population.

**Supplementary Information:**

The online version contains supplementary material available at 10.1007/s12028-023-01700-w.

## Introduction

During the coronavirus disease 2019 (COVID-19) pandemic, a large number of patients required veno-venous extracorporeal membrane oxygenation (VV ECMO) as rescue therapy for acute respiratory distress syndrome (ARDS) refractory to conventional treatment [1].

Neurological complications are common in patients with COVID-19, and different pathophysiological mechanisms have been suggested, including direct central nervous system invasion, activation of coagulative and inflammatory cascades, and systemic hypoxemia [2]. In addition, ECMO may increase the risk of neurological complications, in particular stroke, independently of COVID-19 infection [3, 4].

A recent meta-analysis including 1,322 patients from 12 case series and retrospective cohort studies [1] reported that the prevalence of neurological complications was high in patients with COVID-1 undergoing VV ECMO, suggesting the implementation of neuromonitoring protocols to assess and earlier recognize changes in cerebral dynamics in this population. Noninvasive neuromonitoring systems are widely used in the neurointensive care settings for patients with primary cerebral damage. At present, only a few small studies are available regarding the application of noninvasive neuromonitoring in the general intensive care unit (ICU) population and, in particular, during ECMO, despite preliminary reports suggesting an important role of these tools in the early detection of cerebral complications and potential prediction of outcome [5–7].

In patients with COVID-19, considering the limited resources related to the pandemic, neuromonitoring tools were used in a minority of patients [8]. Among these, Transcranial Doppler (TCD) ultrasonography was the most widely used, as it is a safe, bedside technique that allows for tracking important alterations in cerebral dynamics such as increased noninvasive intracranial pressure. However, no data are reported on this topic in patients undergoing VV ECMO support, especially regarding more advanced TCD-derived parameters such as cerebral autoregulation (CA). Therefore, we performed an observational study intending to assess the cerebrovascular dynamics in patients with COVID-19 undergoing VV ECMO treatment and their association with patients’ ICU mortality.

## Materials and Methods

### Ethics Statement

The study conforms to the Declaration of Helsinki, and the research protocol was approved by the Bioethics Committee at the Medical University in Wroclaw (project “The state of autoregulation of the cerebral circulation in patients with acute respiratory failure in the course of SARS-COV-2”; approval: KB 143/2022 [24.02.2022]), which waived the requirement for informed consent based on the study design. This analysis is reported according to the Guidelines for Strengthening the Reporting of Observational Studies in Epidemiology Statement.

### Aims

This study aimed to describe the cerebrovascular dynamics, in particular CA, using TCD and cerebral biomarkers values, i.e., neuron-specific enolase (NSE), in patients with COVID-19 undergoing VV ECMO treatment and their ability to predict ICU mortality.

### Patient Population

**Fig. 1:**
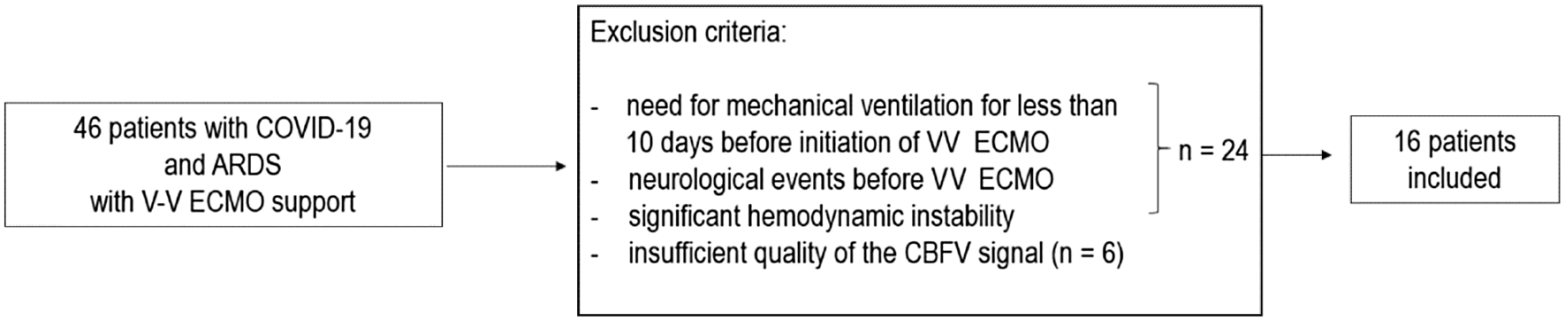
Flow chart of study design based on the recordings captured in patients with a diagnosis of coronavirus disease 2019 (COVID-19) and acute respiratory distress syndrome (ARDS) undergoing veno-venous extracorporeal membrane (VV ECMO) treatment. CBFV, cerebral blood flow velocity

This retrospective, single center, observational study was conducted at the ICU of the University Hospital in Wroclaw from October 2020 to February 2022. Inclusion criteria were adult patients with a diagnosis of COVID-19 by nasal pharyngeal swab for reverse transcriptase-polymerase chain reaction who fulfilled the four following criteria: a diagnosis of severe ARDS as defined by the criteria of the Berlin definition [8] and hypoxemia refractory to conventional treatment who required VV ECMO support; the need for mechanical ventilation for less than 10 days before initiation of VV ECMO; an expected duration of VV ECMO support of at least 72 h; and TCD recordings for at least 20 min with a sufficient quality of the signal after ECMO initiation. The exclusion criteria included previous neurological events and significant hemodynamic instability. The flow chart of the study design is presented in Fig. 1.

### Study Protocol

The collected data included demographic characteristics, clinical scores (Sepsis-related Organ Failure Assessment (SOFA) and Acute Physiology and Chronic Health Evaluation II (APACHE II)), neurological status, physiological parameters on admission; parameters and duration of ECMO support, complications and outcome using Barthel index [9]. SOFA was assessed at 1) admission to the ICU, 2) at the time of TCD measurement, 3) after VV ECMO discontinuation, and 4) after discharge from ICU. VV ECMO support was initiated in cases of severe hypoxic respiratory failure or carbon dioxide retention despite conventional management, including lung-protective mechanical ventilation, prone positioning, and neuromuscular blockade according to local protocols and current guidelines [10]. The decision to start VV ECMO was made by the ECMO team of the ICU of Wroclaw University Hospital based on the Extracorporeal Life Support Organization guidelines [11]. The VV ECMO circuit included The Quadrox-i adult microporous membrane oxygenator (MAQUET Holding BV & Co, KG, Germany) and CardioHelp or Permanent Life Support Set and Rotaflow II Base Unit (MAQUET Holding BV & Co, KG, Germany). An infusion of unfractionated heparin was administered during cannulation and continued for the duration of extracorporeal support aiming at partial thromboplastin time 1.5 times above the normal limit. ECMO parameters were adjusted to the patient’s clinical condition, target pump flow was more than two thirds of the patient’s cardiac output. Multimodal analgosedation was performed in all patients on ECMO based on the Richmond Agitation and Sedation Scale (RASS) to promote ventilatory synchrony and oxygenation and reduce the stress response. Patients were assessed daily for possible ECMO weaning with the use of End of Life Options Act clinical and physiological criteria [11]. Neurological complications were defined as the following: focal neurological deficits, changes in pupil diameter and reactivity, deterioration in the level of consciousness, coma, and changes in the patient’s mental status or occurrence of the ICU-acquired weakness [12] and were assessed daily through physical examination. The management of vasopressors, fluid therapy, steroids and antibiotics, and continuous renal replacement therapy was driven by clinical judgment, and laboratory data were adjusted to the current patient’s condition. Specific COVID-19 treatment during VV ECMO included antiviral drug (Remdesivir), anti-interleukin-6 receptor monoclonal antibodies (Tocilizumab), and extracorporeal cytokine adsorber (Cytosorb) when it was deemed justified. The data collected during VV ECMO comprised arterial blood pressure (ABP), ventilator settings, arterial blood gas parameters, parameters of ECMO and laboratory values of serum biomarkers. Routine blood tests included full blood count, blood chemistry, electrolytes, liver function parameters, renal function parameters, and coagulation markers. Inflammatory-specified markers, included C-reactive protein, procalcitonin, white blood cell count, interleukin-6, albumin-fibrinogen ratio, neutrophil–lymphocyte ratio, and platelet-lymphocyte ratio, were collected at ICU admission. Blood samples for measurement of arterial blood gas parameters, hemoglobin, and hematocrit and NSE analysis were obtained at the same time as TCD measurements.

### TCD Data Acquisition and Parameters

Cerebral blood flow velocity (CBFV) in the middle cerebral artery was measured using a TCD, with a 2-MHz probe (Doppler BoxX; DWL Compumedics Germany GmbH, Singed, Germany). TCD-derived signal was digitized using an analog-to-digital converter and sampled at a frequency of 200 Hz. CBFV was monitored for at least 20 min. ABP was measured via a pressure transducer (Argon Standalone DTX Plus; Argon Medical Devices Inc., Plano, TX) in the radial or femoral artery. ABP was digitized by using an RS-232 serial port and recorded using a laptop computer with Intensive Care Monitoring (ICM+) software (Cambridge Enterprise Ltd, Cambridge, UK). All artifacts were removed manually or by using custom-written algorithms, and further analysis was performed on the representative part of the signals. CA was estimated using a mean flow index (Mxa) as a correlation coefficient between slow-wave oscillations in ABP and CBFV within a 300-s moving time window advanced in 10-s steps [13]. Altered CA was defined as a positive Mxa (Mxa > 0) [14]. Details of the other cerebral hemodynamics formulas derivation are described in previous papers [15–19] and summarized in the Supplementary Data. Based on the model of changes in cerebral arterial blood volume [20–22] and utilizing CBFV and ABP signals we estimated compliance of cerebral arterial bed (C_a_) [20], the resistance of cerebrovascular bed (CVR) [20], the time constant of cerebral arterial bed (τ) [16], critical closing pressure (CrCP) [17], diastolic closing margin (DCM) [23], spectral pulsatile index (sPI) [18], noninvasive intracranial pressure (nICP) [24], and noninvasive cerebral perfusion pressure (nCPP) [25].

### Statistics

The normality of the data was assessed using the Shapiro–Wilk test. Because of the lack of normality distribution for most of the analyzed parameters and limited observations, nonparametric tests were applied. The differences in median values were tested using the Mann–Whitney *U*-test along with an estimation of the Glass rank biserial correlation coefficient as the metric of a size effect (r_G_). Correlation between numerical data was assessed using Spearman’s rank test. A contingency table was used to test the frequency distribution of the variables. The significance of the association between the two kinds of classification was assessed using the Pearson *χ*^2^ test (or Fisher exact test). The receiver operator characteristic curves with area under the curve (AUC) scores were used to determine the cutoff values for biomarkers for the prediction of in-hospital mortality. The level of significance was set at 0.05 in all analyses. Statistical analysis was performed using STATISTICA 13 (Tibco, Palo Alto, USA) and R Statistical Software (v.4.0.2; R Foundation for Statistical Computing, Vienna, Austria). Data are presented as median (first–third quartile) unless indicated otherwise.

## Results

### Patient Characteristics and Clinical Outcomes

Detailed clinical characteristics of the group are presented in Table 1. A total of 16 patients fulfilled the inclusion criteria and were enrolled in the study. The median age was 39 (34–56) years, 14 patients were men, and, in most cases, they were classified as overweighed (six patients) or obese (seven patients). A total of 13 patients had a RASS of − 5, and three patients had a RASS of − 4. All patients in our cohort were not vaccinated against COVID-19. Seven patients died in the ICU. Neurological complications were found in ten patients: unilateral paresis (three patients), ICU-acquired weakness (two patients), anisocoria (two patients), and others in three patients. Brain computed tomography (CT) scan was performed in ten patients and showed the following pathological changes in five patients: subarachnoid hemorrhage in two patients, hypodense focus in one patient, and dilation of Virchow–Robin space (VRs) in two patients. We found that survivors had higher FiO_2_ (*p* = 0.025; r_G_ = 0.51) and lower PEEP (*p* = 0.038; r_G_ = 0.56) in comparison with nonsurvivors. The VV ECMO–related parameters are presented in Table 2.

**Table 1:** Clinical characteristics in the total group of patients with coronavirus disease 2019 (COVID-19)–related acute respiratory distress syndrome (ARDS) requiring veno-venous circuit of extracorporeal membrane oxygenation (VV ECMO)

Parameter	Total group (*N* = 16)
Age [years]	39 (34–56)
Gender: male	14 (88%)
BMI	27 (26–33)
Overweight	6 (38%)
Obesity	7 (44%)
Vaccination against COVID-19	0
SOFA at admission to ICU	9 (7–9)
APACHE II at admission to ICU	12 (11–14)
Comorbidities:	
Hypertension	0
Chronic respiratory disease, COPD or asthma	1 (6%)
Diabetes	2 (13%)
Ischemic cardiomyopathy	0
History of acute brain injury	0
Other	2 (13%)
Treatment, therapy, and drugs	
Richmond score:	
− 4	3 (19%)
− 5	13 (81%)
HFNC	16 (100%)
Prone positioning	16 (100%)
Vasoactive medications	16 (100%)
EF in echocardiography (n = 15)*:	
> = 60%	11 (73%)
< 60%	4 (27%)
Lung tissue affected by inflammation [%]	80 (80–90)
Renal replacement therapy	13 (81%)
Inhaled nitric oxide	4 (25%)
Neuromuscular blocade	8 (50%)
Ketamine	15 (94%)
Opioids	16 (100%)
Midanium	16 (100%)
Propofol	16 (100%)
Outcome	
Neurological complications	10 (63%)
Mortality rate	7 (44%)
Barthel index (*in survivors*)	73 (70–90)
GCS (*in survivors*)	15

Data are presented as median (lower quartile-upper quartile) or as the number of observations (% of the total group).

BMI, body mass index; COVID-19, Coronavirus Disease 2019; SOFA, Sequential Organ Failure Assessment; APACHE II, Acute Physiology and Chronic Health Evaluation II; COPD, chronic obstructive pulmonary disease; GCS, Glasgow Coma Scale; HFNC, high flow nasal cannula; EF, ejection fraction; GCS, Glasgow Coma Scale

*Echocardiography was not performed in 1 case

**Table 2:** Parameters of Veno-venous circuit of extracorporeal membrane oxygenation (VV ECMO) in the total group of patients with coronavirus disease 2019 (COVID-19)–related acute respiratory distress syndrome (ARDS)

Parameter	Total group *N* = 16	Non-survivors *n* = 7	Survivors *n* = 9
Time-related metrics			
Days from the first symptoms to admission to the ICU	12 (9–16)	13 (8–16)	12 (9–16)
Days on MV before VV ECMO	4 (2–8)	4 (1–9)	3 (2–7)
Days on VV ECMO	20 (14–39)	19 (12–39)	28 (16–39)
Days in ICU	42 (22–63)	21 (19–39)	61 (43–63) ^*^
Before VV ECMO			
Lactate [mmol/L]	1.6 (1.3–1.7)	1.7 (1.1–2.2)	1.5 (1.4–1.6)
HCO_3_ [mmol/L]	32.6 (28.4–33.5)	29.4 (23.9–31.5)	33.5 (33.4–34.0)
FiO_2_	1 (0.9–1.0)	0.9 (0.7–1.0)	1.0^*^
PEEP	12 (10–13)	13 (11–14)	12 (10–12) ^*^
PaO_2_/FiO_2_	68.4 (61.1–84.9)	70.2 (52.6–86.0)	67.8 (66.8–79.0)
PaCO_2_ [mm Hg]	51.5 (44.1–61.7)	48.9 (37.9–61.3)	58.6 (44.5–62.0)
PaO_2_ [mm Hg]	66.9 (58.5–69.8)	65.8 (50.8–67.2)	67.2 (66.8–69.9)
pH	7.4 (7.3–7.4)	7.4 (7.3–7.4)	7.4 (7.3–7.4)
During VV ECMO and TCD measurement			
Blood flow [L/min]	4.5 (4.0–4.8)	4.1 (3.7–4.6)	4.6 (4.2–4.9)
Sweep gas flow [L/min]	8.5 (7.0–10.0)	8.5 (7.0–10.0)	10.0 (8.0–10.0) ^*^
PaO_2_ [mm Hg]	72.7 (66.9–75.9)	72.7 (66.9–75.9)	73.7 (68.0–74.8)
PaCO_2_ [mm Hg]	43.0 (40.0–46.1)	43.0 (40.0–46.1)	43.6 (39.5–45.7)
pH	7.4 (7.4–7.5)	7.4 (7.4–7.5)	7.4 (7.4–7.5)

Data are presented as median (lower quartile–upper quartile)

MV, mechanical ventilation; ICU, Intensive Care Unit; VV ECMO, Veno-venous circuit of extracorporeal membrane oxygenation; HCO_3_, calculated concentration of bicarbonate in arterial blood; FiO_2_, fraction of inspired oxygen; PEEP, Positive End-expiratory Pressure; PaO_2_/FiO_2_ ratio, partial pressure of oxygen/fraction of inspired oxygen; PaCO_2_, the partial pressure of carbon dioxide in arterial blood; PaO_2_, the partial pressure of oxygen in arterial blood; pH, acid–base balance of the blood; p-values based on Mann–Whitney test are marked as follows: ***p < 0.001; **p < 0.01; *p < 0.05

### Cerebral Autoregulation

The median Mxa was 0.1 (0.0–0.2) in the total group. Altered CA was found in 12 patients, where Mxa > 0 was observed in about 20% of the TCD recording time*.* The distribution of Mxa values is shown in Supplementary Data. Six out of seven patients who died had also altered CA. Defective CA was a significant predictor of mortality, with a sensitivity of 85.7% and an accuracy of 56.2%. We found that altered CA occurred in four out of six patients with overweight and in six out of seven patients with obesity. Moreover, altered CA occurred in four out of five patients over the age of 50. In addition, three out of five patients with pathological changes in brain CT and six out of ten patients with neurological complications had altered CA.

### Cerebral Hemodynamics

**Fig. 2:**
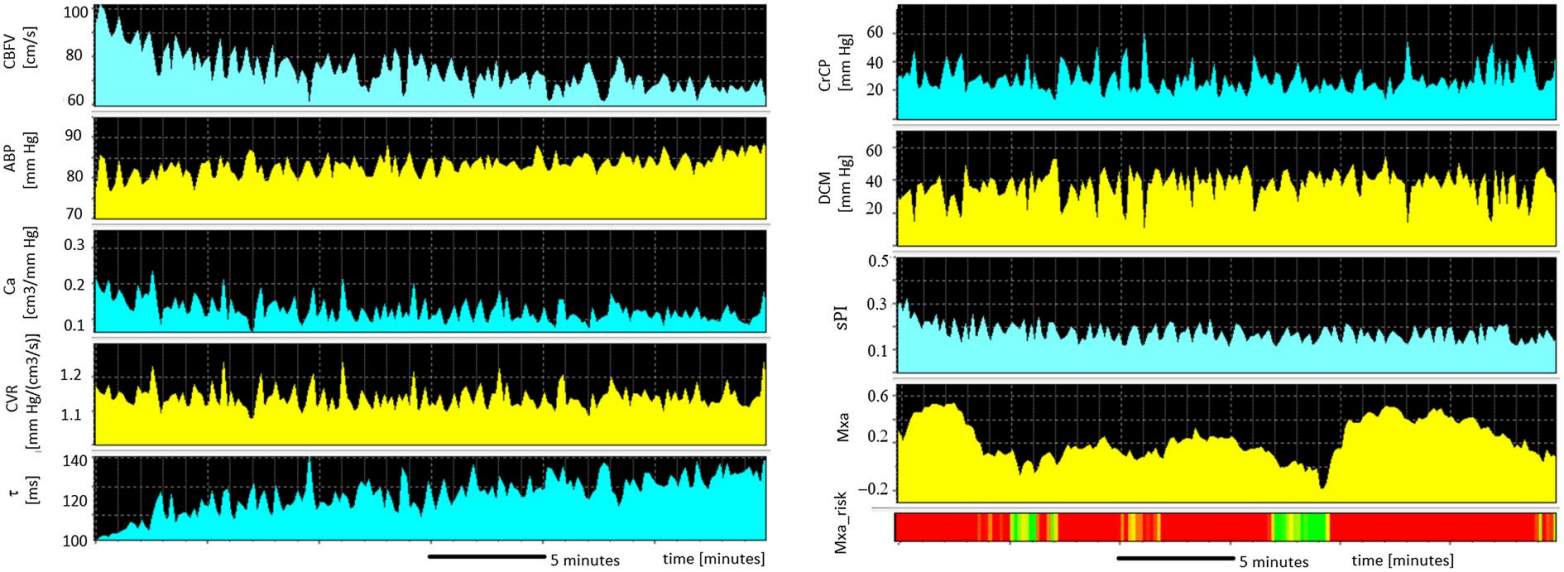
An example of a time course of a 10-s window averaged cerebral hemodynamics parameters and cerebral autoregulation index in 38-year-old patients with a diagnosis of coronavirus disease 2019 (COVID-19) and acute respiratory distress syndrome (ARDS), undergoing veno-venous extracorporeal membrane (VV ECMO) treatment. The following indices are presented (from the left top to the right bottom): ABP, arterial blood pressure; C_a_, compliance of cerebral arterial bed; CBFV, cerebral blood flow velocity; CrCP, critical closing pressure; CVR, cerebrovascular resistance; DCM, diastolic closing margin; Mxa, mean velocity index (index of cerebral autoregulation; Mxa risk means Mxa > 0); sPI, spectral pulsatile index; τ, time constant of cerebral arterial bed

An example of cerebral hemodynamics indices averaged in a 10-s window is presented in Fig. 2. The comparison of physiological and cerebral hemodynamics parameters in survivors vs. nonsurvivors is presented in Table 3. We observed a tendency to longer τ in patients who died in comparison with patients who survived (254.9 [129.5–562.2] ms vs. 169.0 [103.4–212.8] ms; *p* = 0.072; r_G_ = 0.43). Moreover, we observed higher nICP in patients who died in comparison with patients who survived; however, this difference was not statistically significant (22.5 [8.5–27.8] mm Hg vs. 15.5 [11.8–17.9] mm Hg; *p* = 0.169; r_G_ = 0.34). The correlation coefficients between SOFA scores and cerebral hemodynamics are presented in Supplementary Data. In patients who survived, we found a significant, reciprocal correlation between SOFA at admission and C_a_, as well as between SOFA after VV ECMO discontinuation and τ and CrCP. In patients who died, there was a strong correlation between SOFA at admission and cerebrovascular resistance and between CVR, τ, CrCP, and noninvasive cerebral perfusion pressure. See Supplementary Data.

**Table 3:** Physiological parameters and cerebral hemodynamics parameters in the total group of patients with Coronavirus Disease 2019 (COVID-19)-related acute respiratory distress syndrome (ARDS), requiring veno-venous circuit of extracorporeal membrane oxygenation (VV ECMO)

Parameter	Total group (*N* = 16)	Non-survivors (*n* = 7)	Survivors (*n* = 9)
ABP [mm Hg]	85.9 (75.8–93.7)	90.5 (81.9–94.1)	79.4 (74.1–87.9)
AmpABP [mm Hg]	18.9 (17.2–21.0)	19.8 (17.7–21.6)	17.9 (16.9–20.5)
CBFV [cm/s]	48.2 (42.5–64.1)	57.9 (33.3–65.8)	48.0 (43.0–54.8)
AmpCBFV [cm/s]	14.3 (11.2–18.3)	14.2 (11.4–18.6)	14.4 (10.9–17.8)
CVR [mm Hg/(cm^3^∙s)]	1.6 (1.3–1.9)	1.5 (1.2–2.0)	1.7 (1.3–1.8)
C_a_ [cm^3^/mm Hg]	0.11 (0.09–0.13)	0.11 (0.10–0.19)	0.12 (0.07–0.13)
τ [ms]	176.2 (119.5–297.9)	254.9 (129.5–562.2)	169.0 (103.4–212.8)
CrCP [mm Hg]	36.8 (29.0–43.5)	38.5 (26.6–59.4)	35.2 (31.4–40.8)
DCM [ mm Hg]	28.1 (21.0–36.2)	30.0 (17.2–36.3)	26.1 (23.6–36.2)
nICP [ mm Hg]	16.8 (10.2–23.5)	22.5 (8.5–27.8)	15.5 (11.8–17.9)
nCPP [ mm Hg]	65.3 (41.6–74.9)	64.3 (26.1–73.6)	63.3 (49.5–76.3)
sPI [a.u.]	0.2 (0.2–0.3)	0.3 (0.2–0.4)	0.2 (0.2–0.2)

Data are presented as median (lower quartile–upper quartile)

ABP, arterial blood pressure; AmpABP, spectral amplitude of arterial blood pressure; CBFV, cerebral blood flow velocity; AmpCBFV, spectral amplitude of cerebral blood flow velocity; CVR, cerebrovascular resistance; C_a_, compliance of cerebral arterial bed; τ, time constant of cerebral arterial bed; CrCP, critical closing pressure; DCM, diastolic closing margin; nICP, non-invasive intracranial pressure; nCPP, non-invasive cerebral perfusion pressure; sPI, spectral pulsatility index. There were no significant differences in either of the parameters between survivors and non-survivors

### Biomarkers

**Fig. 3:**
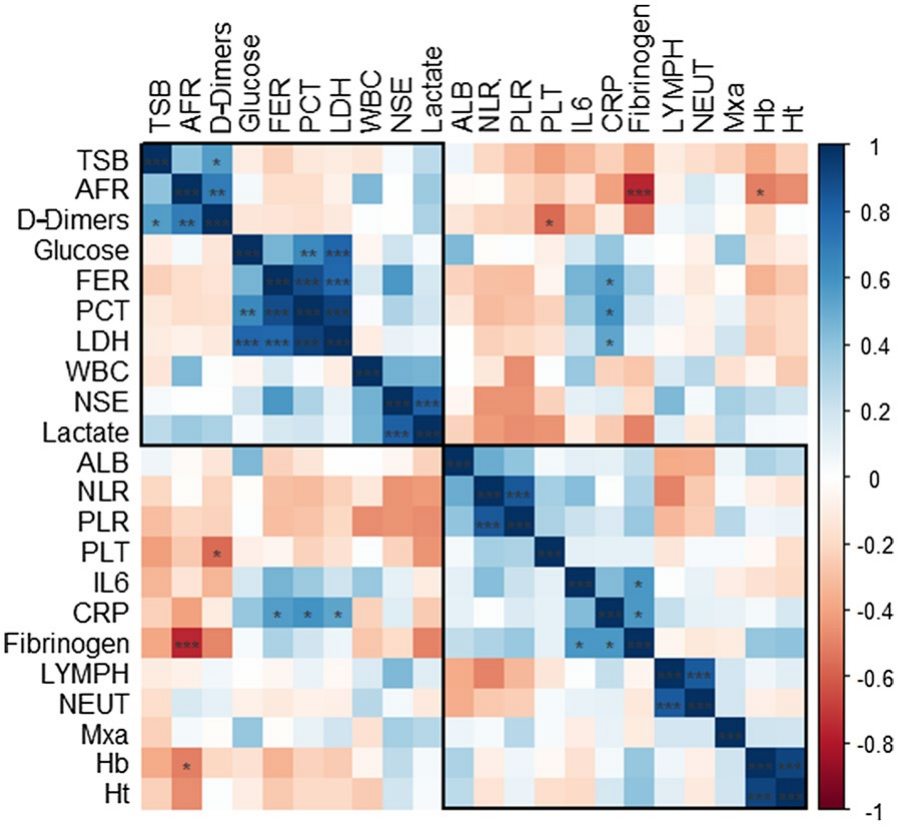
Matrix of Spearman correlation between biomarkers and index of cerebral autoregulation (Mxa) in the total group of patients with a diagnosis of coronavirus disease 2019 (COVID-19) and acute respiratory distress syndrome (ARDS), undergoing veno-venous extracorporeal membrane (VV ECMO) treatment. *p* values for Spearman correlation coefficient are marked as: ***p < 0.001; **p < 0.01; *p < 0.05. Rectangles around the plot of the correlation matrix are based on the results of hierarchical clustering. AFR, albumin-fibrinogen ratio; ALB, albumin; CRP, C-reactive protein; FER, ferritin; Hb, hemoglobin; Ht, hematocrit; IL-6, interleukin 6; Lactate, serum lactate level; LDH, lactate dehydrogenase; LYMPH, lymphocytes; NEUT, neutrophils; NLR, neutrophil–lymphocyte ratio; NSE, neuron-specific enolase; PCT, procalcitonin; PLR, platelet-lymphocyte ratio; PLT, platelets; TSB, total serum bilirubin; WBC, white blood cells

The median values of serum biomarkers concentration in our cohort are presented in Table 4. and the correlation matrix of biomarkers is presented in Fig. 3. For most of the parameters**,** we observed reference values exceeded. Among all analyzed biomarkers, three of them were significant mortality predictors: NSE (cutoff value: 28.9 µg/L; AUC = 0.83, *p* = 0.006), hematocrit (cutoff value: 33.4%; AUC = 0.80, *p* = 0.011), and hemoglobin (cutoff value: 10.9 g/dL; AUC = 0.76, *p* = 0.040).

**Table 4:** Serum biomarkers concentration in the total group of patients with Coronavirus Disease 2019 (COVID-19)-related acute respiratory distress syndrome (ARDS), requiring veno-venous circuit of extracorporeal membrane oxygenation (VV ECMO)

Parameter	Normal range	Total group *N* = 16	Non-survivors *n* = 7	Survivors *n* = 9
IL-6 [pg/mL]	0–5.9	39.7 (21.0–120.0)	95.4 (35.2–136.0)	21.5 (13.7–84.7)
WBC [10^3^/µL]	4.0–10.0	15.9 (13.8–20.6)	19.4 (12.8–24.6)	15.6 (14.6–16.0)
CRP [mg/L]	0–5	106 (71–214)	112 (98–211)	103 (30–216)
PCT [ng/mL]	0–0.05	0.22 (0.15–0.43)	0.20 (0.15–2.76)	0.20 (0.15–0.31)
AFR [a.u.]	–	5.0 (3.5–6.3)	5.0 (3.0–6.0)	5.1 (4.2–6.5)
NLR [a.u.]	–	13.3 (8.1–24.2)	9.0 (4.9–16.9)	18.4 (10.1–25.1)
PLR [a.u.]	–	261 (176–628)	203 (26–643)	297 (258–612)
LDH [U/L]	0–248	659 (523–846)	743 (479–1030)	626 (594–714)
FER [µg/L]	20–250	1399 (516–2264)	1725 (1231–3300)	686 (515–1669)
Fibrinogen [g/L]	2.0–3.9	4.4 (3.9–6.6)	4.6 (3.8–8.1)	4.2 (4.0–5.0)
ALB [g/L]	35–52	27 (25–31)	28 (26–28)	27 (21–31)
D-dimers [µg/mL]	0–0.5	5.8 (2.2–7.2)	5.2 (2.2–7.2)	6.3 (2.2–7.2)
TSB [mg/dL]	0.2–1.2	0.6 (0.5–1.2)	0.8 (0.4–1.3)	0.7 (0.6–1.0)
PLT [10^3^/µL]	140–440	264 (207–313)	231 (200–315)	271 (261–311)
Glucose [mg/dL]	70–99	153 (131–167)	162.0 (152–178)	132 (115–153)
Lactate [mmol/L]	0.5–1.6	1.4 (1.2–1.9)	1.5 (1.2–2.1)	1.2 (0.9–1.8)
LYMPH [10^3^/µL]	1.5–6	1.2 (0.5–2.2)	1.6 (0.5–3.6)	1.1 (0.6–1.3)
NEUT [10^3^/µL]	2.5–6	12.5 (9.7–17.9)	11.8 (8.3–22.2)	12.5 (12.1–13.5)
Hb [g/dL]	14–18	10.8 (9.3–11.0)	10.9 (9.9–11.3)	10.2 (9.0–10.8)
Ht [%]	40–54	33.2 (28.6–34.6)	34.2 (33.2–35.3)	31.4 (27.6–33.2)
NSE [µg/L]	< 18.3	29.1 (19.4–34.9)	38.9 (30.5–72.4)	27.6 (18.2–29.7)

For most of the parameters, we observed reference values exceeded. Data are presented as median (lower quartile–upper quartile)

IL-6, Interleukin 6; WBC, white blood cells; CRP, C-reactive protein; PCT, procalcitonin; AFR, albumin-fibrinogen ratio; NLR, neutrophil–lymphocyte ratio; PLR, platelet-lymphocyte ratio; LDH, lactate dehydrogenase; FER, ferritin; ALB, albumin; TSB, total serum bilirubin; PLT, platelets; Lactate, serum lactate level; LYMPH, lymphocytes; NEUT, neutrophils; Hb, haemoglobin Ht, hematocrit; NSE, neuron-specific enolase; normal range for parameters describe ratio was not determined; p-values based on Mann–Whitney test are marked as follows: ***p < 0.001; **p < 0.01; *p < 0.05

### Correlation Between CA and Biomarkers

The correlation matrix of biomarkers vs. Mxa is presented in Fig. 3. We found a significant relationship between increased levels of NSE (NSE > 28.9 µg/L) and altered CA: *χ*^2^ = 6.24; *p* = 0.035; φ = 0.69. Altered CA was found in all patients with abnormal NSE.

## Discussion

The main findings of this study can be summarized as follows:

1. Altered CA is common in patients with COVID-19 undergoing VV ECMO, and it is a predictor of mortality with good sensitivity.
2. NSE is a significant predictor of mortality, and a significant relationship exists between increased levels of NSE and altered CA.
3. Considering cerebrovascular parameters, patients who survived had lower nICP and prolongated τ in comparison with patients who died, although those findings did not reach statistical significance.

To our knowledge, this is the first study evaluating advanced cerebral hemodynamics in a population of patients with COVID-19 undergoing VV ECMO and exploring the relationship between CA and biomarkers. These preliminary data highlight the importance of the use of neuromonitoring tools, and, in particular, TCD in the management of patients with COVID-19 undergoing ECMO with no primary brain injury but who are at high risk of neurological complications.

The importance of CA in patients with brain injury has been importantly demonstrated over the last decades [26–29], suggesting that impaired CA is an important determinant of secondary brain damage and therefore of outcome. More recently, the application of neuromonitoring tools able to assess CA has been applied in a variety of situations outside the neurocritical care settings. A recent systematic review found 22 articles exploring the role of monitoring CA in the perioperative settings, in ICU patients with sepsis, and in the pediatric population, suggesting that even in patients without brain injury, altered CA may result in increased mortality [30]. In addition, disturbed CA was found as a common event, as it is reported in about 50% of patients in the general ICU [31].

The research of Bombardieri et al. [32] suggests that following the initiation of VV ECMO, P_a_CO_2_ and mean arterial pressure rapidly decrease, resulting in decreased CBFV. In our study, we found an altered CA in 12/16 patients. This is probably related to the specific group of patients that we explored who are at high risk of cerebral derangement [33]. The ECMO itself, despite being a life-saving technique that is widely used in centers throughout the world, may lead to altered cerebral hemodynamics, particularly affecting cerebral blood flow regulation [34] and metabolic function [35]. The unknown individual optimal blood pressure and impaired CA may lead to both hypoperfusion and cerebral hyperperfusion resulting in brain parenchyma damage. In our group of patients, altered CA was found in six out of seven who died. However, most of those patients died as a result of sepsis-related multiorgan failure. Therefore, we believe that sepsis or developing sepsis-associated encephalopathy was the probable cause of cerebral blood flow autoregulation disorders [36–38].

Battaglini et al. [2] showed that neurological complications occurred in 47/94 (50%) of critically ill patients with COVID-19 receiving invasive mechanical ventilation in the ICU. The important finding was that the magnitude of the inflammatory response and the severity of respiratory impairment may strongly affect the occurrence of neurological complications in COVID-19. Therefore, noninvasive neuromonitoring during ICU stay may be helpful to detect cerebrovascular alterations earlier. According to the Extracorporeal Life Support Organization database, with more than 6,200 cases of patients under VV ECMO, the fraction of patients with at least one neurological injury is between 7.2% for single-line ECMO mode to 7.7% for dual-line mode [39]. Another study based on 135 patients undergoing VV ECMO showed that 14.1% of them had a neurologic injury [40]. An earlier study on VV ECMO patient populations indicated that neurological events occurred even in 50% of patients who received VV ECMO [41]. However, these discrepancies between the studies might be explained by indications for ECMO, use of anticoagulation, age, or even different policies to obtain cerebral CT scan [39]. In our study, neurological complications were reported in 10 patients. Among 12 patients with altered CA, six of them demonstrated neurological complications. In the group of four patients with neurological changes, without CA disorders, subarachnoid hemorrhage was found in one patient, unilateral paresis was found in one patient, and widening of the VRs on the CT scan was found in two patients, in which infected VRs are accompanied by inflammatory or metabolic diseases (cerebral small vessel disease) [42]. Interestingly, in the study of Tian et al. [43] on the pediatric population, it was found that high degrees of CA impairment during ECMO were indicative of severe neuroimaging abnormalities. However, the relationship between the alterations in CA and neurological changes was not the main goal of this study.

In previous research on a cohort of patients with COVID-19 undergoing neuromonitoring, increased ICP estimated noninvasively was common, affecting 19% of the overall population, and those with increased ICP had a longer ICU stay. Brasil et al. [33] also found that intracranial compliance and impairment of cerebrovascular hemodynamics are often present in COVID-19 severe illness and could accurately predict an early poor outcome. Moreover, they observed an increase in estimated ICP within normal ranges after ECMO implementation, as a result of reduced jugular drainage after cannulation. These data suggest that this group of patients is particularly susceptible to cerebrovascular alterations for different pathophysiological mechanisms related to the virus itself, but also to systemic complications such as severe respiratory failure and their management, i.e., lung-protective strategies, which can increase intrathoracic and ICP [2]. In our study, we found higher values of noninvasive ICP in the nonsurvivors compared with survivors (although this difference did not reach a statistical significance), which is consistent with previous studies [44]. Moreover, we found longer τ values, which represents the time constant of the cerebral arterial bed and is an index of altered brain dynamics, in nonsurvivors [45]. A prolongation of the τ in nonsurvivors may be related to significantly higher sweep gas flow. In a previous study, a significant increase in the time constant of cerebral circulation was found during hypocapnia after severe traumatic brain injury [46].

The importance of the evaluation of cerebral parameters in this population is also highlighted by the fact that values of NSE were significantly correlated with altered CA. NSE is a specific biomarker for cerebral disease staging and monitoring, with important prognostic value [47, 48]. NSE is known to promote the synthesis of proinflammatory mediators. Its elevated levels in patients with COVID-19 support the hypothesis of cerebral involvement in this pathological condition, with inflammatory invasion and neuronal injury, particularly in patients with neurological symptoms [47]. Interestingly, we found that both high NSE and altered CA are predictors of short-term mortality in our population. Although our data are just preliminary, this suggests that the use of neuromonitoring tools and biomarkers could help in the multimodal assessment of cerebral function and prognosis in this population, and effort should be made to implement them as a routine part of the clinical practice.

### Limitations

This study presents several limitations that need to be mentioned. Firstly, the study was performed on a limited number of patients with ARDS due to COVID-19 requiring VV ECMO, and we have not investigated whether observed changes are caused by VV ECMO itself. Secondly, we did not analyze the pathophysiology of the relationship between CA and neurological complications because they were too heterogeneous in terms of type and severity, and there was no confirmation using magnetic resonance imaging. TCD-based CA assessments were performed within the first 48 h after VV ECMO connection. In this analysis, we chose the first TCD recording in each patient; however, the period between starting VV ECMO and TCD measurement may differ among patients. Brain CT scan was not routinely performed and was ordered in cases in which neurological deterioration occurred or when clinically reasonable. Therefore, there is a limited time correlation between those two examinations. Lastly, we used Mxa > 0 for a threshold value indicating altered CA; however, in the literature, there is a high variability of the possible thresholds referring to impaired functional CA both in healthy adults as well as in critically ill patients. Moreover, it is well known that pharmacological agents affect CA and metabolism variably [49]. As such, these findings should be considered preliminary. Further prospective research in a larger cohort of patients from the multicenter database or in an alternate institution is warranted to confirm the observations presented in this article.

## Conclusions

Patients with COVID-19–related ARDS requiring ECMO are likely to have altered CA and are also more likely to have elevated NSE levels. CA was correlated with NSE values in this population. Further studies are warranted to support the use of advanced neuromonitoring in this population for the early detection of brain injury and prediction of clinical outcomes.

## Supplementary Information

Below is the link to the electronic supplementary material.

Supplementary file1 (DOCX 58 KB)

Supplementary file2 (DOCX 15 KB)

Supplementary file3 (DOCX 49 KB)
