# Supplementary material for: Cerebral Autoregulation, Cerebral Hemodynamics, and Injury Biomarkers, in Patients with COVID-19 Treated with Veno-Venous Extracorporeal Membrane Oxygenation
Source: Neurocrit Care. 2023 Mar 22;39(2):425–35. doi: 10.1007/s12028-023-01700-w (PMC10033181; doi:10.1007/s12028-023-01700-w)
Supplement: Supplementary file 1 — Supplementary file1 (DOCX 58 KB) [file 12028_2023_1700_MOESM1_ESM.docx]

Supplementary Data

Cerebral hemodynamics parameters

**Cerebral Arterial Blood Volume (C_a_BV)**

According to the formula proposed by Avezaat and van Eijndhoven ^1^ the changes in cerebral arterial blood volume (∆C_a_BV) are the effect of the transient interaction between changes in cerebral blood inflow (CBF_in_) and cerebral blood outflow (CBF_out_):

$\Delta C_{a}BV(t)=\int_{t_{0}}^{t} (CBF_{\mathrm{in}}(s)-CBF_{\mathrm{out}}(s))ds$ (1)

where *t_0_* is the beginning, *t* is the end of a single cardiac cycle and *s* is the variable of integration.

Under the assumption that CBF_out_ has a low pulsatility in comparison to CBF_in_, the former component of the equation could be expressed as a mean arterial inflow (CBF_a_). Assuming constant cross-sectional area (S_a_) of the insonated artery and a finite sampling frequency of cerebral blood flow velocity (CBFV) eq. 1 can be expressed as:

$\Delta C_{a}BV(n)=\sum_{i=1}^{n} (CBFV_{a}(i )-meanCBFV(i))\Delta t(i)\cdot S_{a}{[cm}^{3}]$ (2)

Note that for further calculation of cerebral hemodynamics indices ∆C_a_BV was divided into S_a_; thus, the units of ∆C_a_BV and its amplitude is [cm].

**Compliance of Cerebral Arterial Bed (C_a_)**

The compliance of the cerebral arterial bed (C_a_) was defined as a ratio between the amplitude of ∆C_a_BV (Amp∆C_a_BV) and the amplitude of arterial blood pressure (AmpABP) ^2^. Both amplitudes were estimated using a Fast Fourier Transform:

$C_{a}=\frac{\text{Amp}\Delta C_{a}\text{BV}}{\text{AmpABP}} [\frac{\mathrm{cm}}{mm Hg}]$ (3)

**Cerebrovascular resistance (CVR)**

Cerebrovascular resistance (CVR) was estimated as a ratio between mean ABP and mean CBFV according to following formula ^3^:

$CVR=\frac{\mathrm{meanABP}}{\mathrm{meanCBFV}}\left[ \frac{\mathrm{mmHg}\cdot s}{\mathrm{cm}} \right]$ (4)

**Time Constant of Cerebral Arterial Bed (τ)**

The time constant of cerebral arterial bed (τ) was calculated, by analogy to electric RC circuit, as a product of C_a_ and CVR. Note, that this parameter is independent from S_a_ of vessel and expressed in seconds.

$\tau=C_{a}\cdot\mathrm{CVR} [s]$ (5)

**Critical closing pressure (CrCP)**

Critical closing pressure (CrCP) reflects ABP at which brain vessels collapse causing
a cessation of cerebral blood flow ^4^. It was expressed according to multi-parameters formula proposed by Varsos ^4^ as:

$CrCP=meanABP- \frac{\mathrm{meanABP}}{\sqrt{\left( C_{a}\cdot CVR\cdot HR\cdot2\pi\right)^{2}+1}} [mm Hg]$ (6)

**Diastolic Closing Margin (DCM)**

Diastolic closing margin (DCM) is a difference between diastolic ABP (dABP) and CrCP ^5^.

$DCM=mean dABP-CrCP [mm Hg]$ (7)

This parameter is based on the observation that dABP less than CrCP results in a cessation of cerebral blood flow, observed as zero diastolic CBFV ^5^.

**Spectral pulsatile index (sPI)**

Spectral pulsatility index (sPI) is defined as the ratio between spectral pulse amplitude of CBFV (AMP_CBFV_) and mean CBFV as follows ^6^:

$sPI=\frac{{AMP}_{CBFV}}{meanCBFV} [a.u.]$ (8)

AMP_CBFV_ was estimated using a Fast Fourier Transform as the amplitudes of fundamental components of CBFV.

**Non-invasive intracranial pressure (nICP) and cerebral perfusion pressure (CPP)**

Non-invasive intracranial pressure (nICP) was estimated according to formula proposed by Czosnyka as follows ^7^:

$nICP=meanABP\cdot\left( \frac{1-mean dCBFV}{mean CBFV} \right)-14 [mm Hg]$ (9)

where dCBFV is the diastolic cerebral blood flow velocity.

Then non-invasive cerebral perfusion pressure (nCPP) was deifned as a difference between mean ABP and mean non-invasive ICP:

$nCPP=mean ABP-mean nICP$ (10)

**References**

1. Avezaat CJJ, van Eijndhoven JHM. The role of the pulsatile pressure variations in intracranial pressure monitoring. Neurosurg Rev 1986;9(1–2):113–20.

2. Kim D-J, Kasprowicz M, Carrera E, et al. The monitoring of relative changes in compartmental compliances of brain. Physiol Meas 2009;30(7):647–59.

3. Czosnyka M, Piechnik S, Richards HK, Kirkpatrick P, Smielewski P, Pickard JD. Contribution of mathematical modelling to the interpretation of bedside tests of cerebrovascular autoregulation. J Neurol Neurosurg Psychiatry 1997;63(6):721–31.

4. Varsos G v., Richards H, Kasprowicz M, et al. Critical closing pressure determined with a model of cerebrovascular impedance. Journal of Cerebral Blood Flow & Metabolism 2013;33(2):235.

5. Varsos G v., Richards HK, Kasprowicz M, et al. Cessation of diastolic cerebral blood flow velocity: the role of critical closing pressure. Neurocrit Care 2014;20(1):40–8.

6. de Riva N, Budohoski KP, Smielewski P, et al. Transcranial doppler pulsatility index: What it is and what it isn’t. Neurocrit Care 2012;17(1):58–66.

7. Czosnyka M, Matta BF, Smielewski P, Kirkpatrick PJ, Pickard JD. Cerebral perfusion pressure in head-injured patients: a noninvasive assessment using transcranial Doppler ultrasonography. J Neurosurg 1998;88(5):802–8.
