# Supplementary material for: Cerebral Autoregulation, Cerebral Hemodynamics, and Injury Biomarkers, in Patients with COVID-19 Treated with Veno-Venous Extracorporeal Membrane Oxygenation
Source: Neurocrit Care. 2023 Mar 22;39(2):425–35. doi: 10.1007/s12028-023-01700-w (PMC10033181; doi:10.1007/s12028-023-01700-w)
Supplement: Supplementary file 2 — Supplementary file2 (DOCX 15 KB) [file 12028_2023_1700_MOESM2_ESM.docx]

Supplementary Table 1. Relationship between Sepsis-related Organ Failure Assessment (SOFA), assessed (1) at admission, (2) during Transcranial Doppler ultrasonography, (3) after a veno-venous circuit of extracorporeal membrane oxygenation (VV ECMO) discontinuation and (4) at discharge from ICU, and cerebral hemodynamics parameters in the total group and survival-based subgroups of patients with a diagnosis of Coronavirus Disease 2019 (COVID-19) and severe acute respiratory distress syndrome (ARDS).

|  | CVR | C_a_ | τ | CrCP | DCM | nICP | nCPP | sPI |
| --- | --- | --- | --- | --- | --- | --- | --- | --- |
|  | Survivors (n = 9) | | | | | | | |
| SOFA (1) | 0.52 | **-0.89^***^** | -0.60 | -0.49 | 0.40 | 0.03 | 0.01 | -0.17 |
| SOFA (2) | 0.19 | -0.35 | -0.03 | 0.03 | 0.60 | 0.19 | 0.47 | -0.19 |
| SOFA (3) | 0.07 | -0.58 | **-0.74^*^** | **-0.86^**^** | 0.70 | 0.31 | -0.17 | -0.28 |
| SOFA (4) | -0.20 | -0.16 | -0.05 | -0.11 | 0.53 | 0.09 | 0.48 | 0.20 |
|  | Non- Survivors (n = 7) | | | | | | | |
| SOFA (1) | **0.81^*^** | 0.28 | **0.88^**^** | **0.99^***^** | -0.66 | 0.66 | **-0.76*** | 0.70 |
| SOFA (2) | 0.45 | 0.03 | 0.45 | 0.41 | -0.30 | 0.30 | -0.68 | 0.13 |
| SOFA (3) | 0.10 | 0.10 | 0.50 | 0.50 | -0.10 | 0.10 | -0.20 | 0.30 |
| SOFA (4) | 0.10 | 0.10 | 0.50 | 0.50 | -0.10 | 0.10 | -0.20 | 0.30 |

Abbreviations: ICU – Intensive Care Unit, SOFA(1),(2), (3), (4)- Sepsis-related Organ Failure Assessment (SOFA) assessed (1) at admission to the ICU, (2) during Transcranial Doppler ultrasonography, (3) after a veno-venous circuit of extracorporeal membrane oxygenation (VV ECMO) discontinuation and (4) at discharge from ICU; CVR–cerebrovascular resistance, C_a_-compliance of cerebral arterial bed, τ–time constant of cerebral arterial bed, CrCP–critical closing pressure, DCM– diastolic closing margin, nICP–non-invasive intracranial pressure, nCPP- non-invasive cerebral perfusion pressure, sPI–spectral pulsatility index; p-values for Spearman correlation coefficient are marked as: ***p<0.001; **p<0.01; *p<0.05
