# Supplementary material for: Cerebral Autoregulation, Cerebral Hemodynamics, and Injury Biomarkers, in Patients with COVID-19 Treated with Veno-Venous Extracorporeal Membrane Oxygenation
Source: Neurocrit Care. 2023 Mar 22;39(2):425–35. doi: 10.1007/s12028-023-01700-w (PMC10033181; doi:10.1007/s12028-023-01700-w)
Supplement: Supplementary file 3 — Supplementary file3 (DOCX 49 KB) [file 12028_2023_1700_MOESM3_ESM.docx]

**
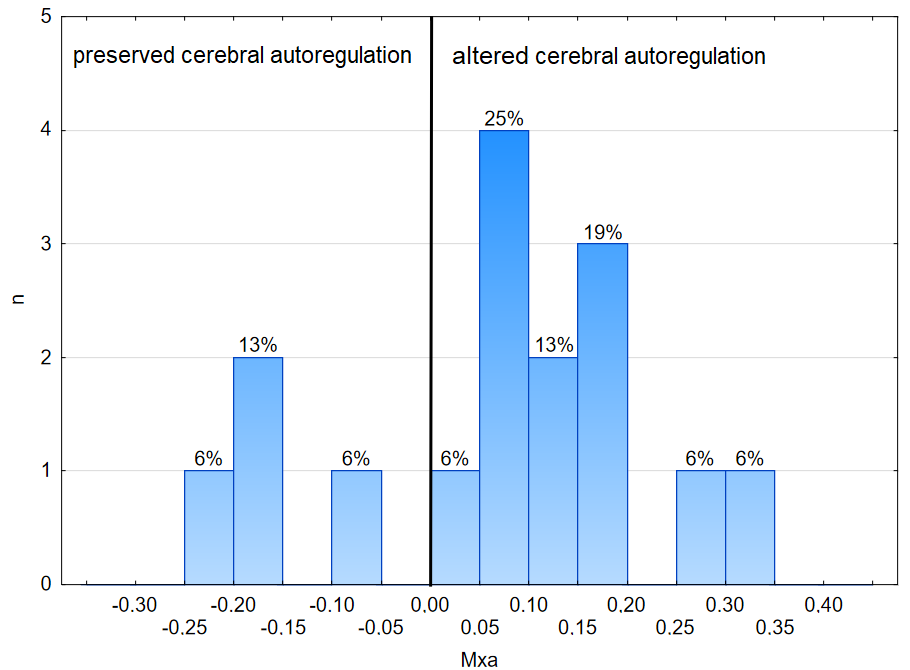
Supplementary Figure 1**

The distribution of mean velocity index (Mxa) in a total group. The positive value of Mxa indicates altered cerebral autoregulation, whereas the negative value reflects preserved cerebral autoregulation.
